# Sociable Weavers Increase Cooperative Nest Construction after Suffering Aggression

**DOI:** 10.1371/journal.pone.0150953

**Published:** 2016-03-16

**Authors:** Gavin M. Leighton, Laura Vander Meiden

**Affiliations:** 1 Cornell University, Cornell Laboratory of Ornithology, Ithaca, NY, United States of America; 2 University of Miami, Department of Biology, Coral Gables, FL, United States of America; Liverpool John Moores University, UNITED KINGDOM

## Abstract

The major transitions in evolution rely on the formation of stable groups that are composed of previously independent units, and the stability of these groups requires both cooperation and reduced conflict. Conflict over group resources may be common, as suggested by work in both cichlids and humans that has investigated how societies resolve conflict regarding investment in group resources, i.e. public goods. We investigated whether sociable weavers (*Philetairus socius*) use aggressive behaviors to modulate the cooperative behavior of group mates. We find that the individuals that build the communal thatch of the nest, i.e. the individuals most at risk of exploitation, are the most aggressive individuals. We show that individuals that invest in interior chamber maintenance, possibly a more selfish behavior, suffer relatively more aggression. After suffering aggression individuals significantly increase cooperative construction of the communal nest thatch. We show that cooperative individuals target aggression towards selfish individuals, and the individuals suffering aggression perform cooperative behaviors subsequent to suffering aggression. In addition to other evolutionary mechanisms, these results suggest that aggression, possibly via the pay-to-stay mechanism, is possibly being used to maintain a public good.

## Introduction

The major transitions in evolution often rely on the maintenance of communal resources that allow individuals to coalesce in both space and time. The stability of these groups requires both cooperation and limited conflict within the group [1]. Although elevated relatedness can reduce conflict within a group [2], there will still be conflict in groups when the fitness of individuals is not perfectly aligned [3, 4]. Thus conflict is prevalent, and is found in microbial, insect, mammal, fish, and bird societies [5–11], especially in regards to reproductive share within groups [12, 13]. However, there is likely also conflict over investment in cooperative behaviors that produce a resource that benefits the entire group, i.e. a public good [14]. Public goods are common in many societies; for example, groups of microbes often exude chemicals that facilitate resource acquisition [15], and certain species of spider rely on communal webs for prey capture [16]. Similarly, several vertebrate societies rely on communal burrows for the safety and maintenance of the group [17, 18]. Despite the importance of public goods for the stability of many animal societies [19], relatively less work has investigated intra-group conflict over cooperative investment in public goods compared to investment in reproduction, though work in *Neolamprologus pulcher* has investigated how helpers contribute to territory maintenance and defense [20] and work in *Polistes fuscatus* has investigated how conflict over individual investment changes with worker value [21].

While public goods often underlie the stability of a group they are simultaneously at risk of being over-exploited by selfish individuals within the group [22], and over-exploitation can lead to a collapse of the resource, i.e. a ‘tragedy of the commons’ [14]. Several mechanisms are thought to prevent a tragedy of the commons; for instance, kin-directed benefits are considered important for the maintenance of groups and group resources [10, 23]. Group resources may also be stabilized by aggression or coercion. For instance, in humans and in *N*. *pulcher*, control of maintenance and consumption of public goods is often directed via top-down mechanisms or enforcement between parties [14, 24, 25]. Both punishment [26] and pay-to-stay [20] are evolutionary mechanisms where aggression is used to coerce cooperative investment in group resources. Indeed, coercive mechanisms can lead to increased cooperative investment investment in public goods compared to kin selection [26]. However, in the case of kin directed benefits, there often needs to be additional behavioral mechanisms to prevent exploitation of the communal resource. To protect investment in public goods individuals may utilize aggressive behaviors to induce cooperation and prevent exploitation [27]. Indeed, there is evidence in birds [7], mammals [12], and multiple insects [28, 29] that aggression is used to regulate reproductive behavior of subordinate group mates. Recent evidence in fish suggests that aggression can be used to induce high levels of cooperative investment into a communal resource [26]. Outside of this recent study [26], relatively little work has investigated the generality of the idea of whether aggression is used to regulate cooperative investment into a communal resource.

We therefore investigated how aggression modulates cooperative nest construction in sociable weavers (*Philetairus socius*). Sociable weavers are a useful system for studying social evolution because individuals live in communal nests with multiple chambers present within the nest [30, 31]. Recent evidence suggests that cooperative nest construction in sociable weavers is maintained, at least in part, by the acquisition of indirect benefits [32, 33]. Since the communal nest is a public good [34–36], the benefits of nest building may be exploited by others in the nest [33]; cooperative individuals may still need to limit exploitation. Sociable weavers may rely on aggression to limit exploitation, and cooperative nest construction may therefore be maintained via multiple mechanisms in sociable weavers.

Sociable weaver nests are maintained via two disparate routes. First, individuals can maintain the chambers they roost in at night, with individuals showing fidelity to specific chambers [35]. Second, individuals can insert items into the nest thatch, from here on referred to as the communal thatch, and spend time re-weaving materials already in the thatch [37]. The first route, i.e. maintaining individual chambers, may be relatively selfish because individuals can monopolize the benefits of the behavior. We measured both forms of nest construction as well as aggressive behaviors that have been defined for sociable weavers [31].

We derive predictions based on theoretical investigation of how aggression can be used to regulate cooperative behaviors in general [38]. The original model by Clutton-Brock and Parker [38] predicts that cooperative individual A suffers a fitness cost when performing a cooperative behavior that is exploited by uncooperative individual B. In the second step individual A again suffers a fitness cost to inflict a relatively high fitness cost to individual B. In response to the punishment, individual B switches behavior and performs a cooperative behavior at a fitness cost to itself that benefits individual A. We used this model as a tentative framework for exploring aggression and cooperation in sociable weavers. We assume that sociable weavers that cooperatively build the communal thatch of the nest are at risk of exploitation, and therefore the most cooperative individuals should be selected to protect their investment in the communal resource. We predict that the most cooperative individuals will be the most aggressive so as to limit exploitation. We predict that individuals that do not contribute to construction of the communal thatch will be preferentially attacked since these individuals are exploiting the effort of other weavers, and that individuals that suffer aggression will increase cooperative output after suffering aggression.

## Materials and Methods

### Study Site and Species

All research was performed following the permits: permit 1629/2011 from the Ministry of Environment and Tourism from Namibia and IACUC permit 12–098 from the University of Miami. Sociable weavers are colonial passerines (24–30 g) that live in the semi-arid savannahs of Southwestern Africa (primarily Namibia and South Africa). Individuals live in colonies and build a communal, perennial nest [30] with multiple chambers where individuals roost at night. These chambers provide thermal benefits [34] and individuals repeatedly return to the same chambers to roost over multiple nights [35]. Sociable weavers are cooperative breeders that use nest chambers as breeding chambers [39], and males are more related to colony mates than females [32, 40]. Most items inserted into the nest are twigs, *Stipagrostis spp*. grass, and softer materials are used as nest chamber lining [30]. Sociable weavers feed on a diet of insects and seeds [30], with individuals from different nests differing in dietary profiles [41].

We studied three sociable weaver nests at the Brink Research Site in central Namibia between May and June 2014 with all three nests located in the same tree. Given the spatial proximity of nests there is concern regarding pseudoreplication. However, previous work on sociable weavers shows that sociable weavers build at only one nest [37] and often build in one section of the single nest they inhabit [32]. Similarly, relatives often occupy spatially distinct sections of a nest and are unlikely to have multiple relatives at other nests [32]. However, given that the nests were in the same tree we interpret all results with caution. We captured individuals using mist-nets and placed individual-specific color leg bands on 83 individuals at three nests (n = 31, 39, 13, for the three nests). Based on visual sightings of unbanded birds we estimate that ~70% of the birds were banded in across all three nests. Observation bouts of three hours were performed in the morning (08:30–11:30) or in the afternoon (13:30–16:30) following [33]. We performed observations during the months that are the beginning of the austral winter, and sociable weavers typically do not breed during this season. However, we did hear two nest chambers that had nestlings at the start of the observation period, though both sets of clutches failed by the third week. We recorded the following morphological measurements for each individual: weight to the nearest 0.1 g, wing length to the nearest 0.5 mm, and tarsus length, beak depth, and beak width to the nearest 0.01 mm. We regressed weight on tarsus to estimate body condition. We also categorized age as adult or juvenile based on plumage [30].

Since sociable weaver adults are monomorphic in terms of plumage we determined the sex of individuals genetically. We drew ~40 μL of blood from the brachial vein and stored the blood in lysis buffer. We extracted DNA following the protocol of Bush, Vinsky [42] using Qiagen DNA kits. We ran multiplex PCR amplification with the P0, P2, and P8 primers [43] to determine the sex of individuals in the study. We were not able to successfully amplify 24 of the 83 individuals bands despite 5+ reactions where we varied PCR reaction concentrations or thermal cycler settings. The DNA did not seem to be degraded based on gel imaging of whole nuclear DNA and all concentrations of DNA for failed individuals was > 5 μg/ml, thus we are uncertain of why these reactions failed.

### Behavioral Observations

We observed behavior from two observation blinds that were set up ~5 meters away from the nest and set at 180° from each other. The observation blinds were established 5 days prior to observation so that individuals acclimated to the presence of the blinds. We used scan sampling to observe individuals for a total of 248 hours and recorded several behaviors as they were performed by individuals at the nest. These observation methods follow those in both van Dijk, Kaden [32] and Leighton, Echeverri [33]. We recorded the total number of items individuals inserted into the communal thatch and the amount of time individuals spent weaving material into the communal thatch. We also recorded the number of items individuals brought into an internal nest chamber for chamber maintenance. We did not record time in nest chamber since it is ambiguous whether individuals spent all the time in a nest chamber weaving material into the chamber wall. Finally, we measured chasing behavior, a previously described behavior that is considered aggressive in sociable weavers [31]. Although individuals may be chased away from the nest temporarily, individuals do not seem to be permanently evicted as they are observed at the nest the following day or on the same day (G.M. Leighton, pers. obs.). For each chase, we recorded the individual that initiated the chase and the individual that was being chased where possible. Typically, the result of a chase is an individual is chased away from the nest and does not return for a variable amount of time (Leighton, pers. obs.). If a chase included an unbanded individual we still recorded the other party in the aggressive encounter. Although other aggressive behaviors have been documented in sociable weavers [44], these aggressive behaviors were recorded at artificial feeding stations and we observed none of the other physical attack behaviors recorded in that study.

### Statistical Analysis

As the dependent variables in the data were count data and displayed increased variance at higher values, we employed generalized linear mixed models (GLMM) with a negative binomial error structure and a log-link function [45]. We built these models in R [46], version 3.1.2, using the package lme4 [47]. In all of the models we assigned individual, nest, and date as random factors. We then used p-values to assess regression coefficients and whether they differed significantly from zero. The models were fit using maximum likelihood with a Laplace approximation as this performs better than other alternatives [48]. We constructed two main models. First, the the independent variables: condition; wing length; tarsus; beak depth; beak width; items inserted into the thatch; items inserted into the nest chamber; age class; and sex; were used to predict the level of aggression. And second, the same independent variables as in the first model were used to predict the level of aggression suffered. We only used items inserted into the thatch as a predictor and not time weaving items into the thatch as these two variables are correlated and would result in collinearity in the model. We chose items inserted into the thatch over time spent weaving as the number of items inserted into the thatch is more repeatable [33]. To further investigate the variables associated with nest construction we regressed the number of items an individual inserted into the nest thatch on the number of items inserted into a nest chamber. We used mixed models to determine how the nest construction behavior of individuals changed before suffering aggression and after suffering aggression. We estimate R^2^ values for each full model according to Nakagawa and Schielzeth [49] and implemented in R by Lefcheck and Cassallas [50]. We calculated the repeatability of each of the behaviors we measured using the “rptR” function in R, which is based on the suggested repeatability described by Nakagawa and Schielzeth [51]. Repeatability was calculated for individuals and the unit of observation is each 3-hour observation block following [33]. Specifically, the cooperative output of individuals is compared across the 3-hour observation blocks. Since we have missing values for sex we ran all multivariate analyses on the reduced subset where we have information for sex of the individuals. If sex was not significant we then removed it from the analysis and analyzed the larger dataset by including individuals whose sex was not identified.

In our dataset we had 10 juveniles (~12% of the individuals). Of the individuals that we could successfully determine sex (see above), we identified 24 males (~41%) and 34 females (~59%). 11 of the 34 females were never observed building the thatch of their nest, and 9 of the 24 males were never observed building the thatch of their nest. 19 of the 34 females were never observed chasing a colony mate (our measure of aggression), while 13 of the 24 males were never observed chasing a colony mate. Males were more aggressive than females on average, performing ~1.5 more chases than females. In total we observed 342 chases and were able to identify the individual being chased in 73 cases. As birds are being chased away from their nest this led to a subset of chases where we could observe both the aggressive individual and the individual being chased from the nest. In the age classes, none of the juveniles (0/10) were observed chasing colony mates. Interestingly, 9 of the 10 juveniles were never chased by colony mates.

## Results

**Fig 1:**
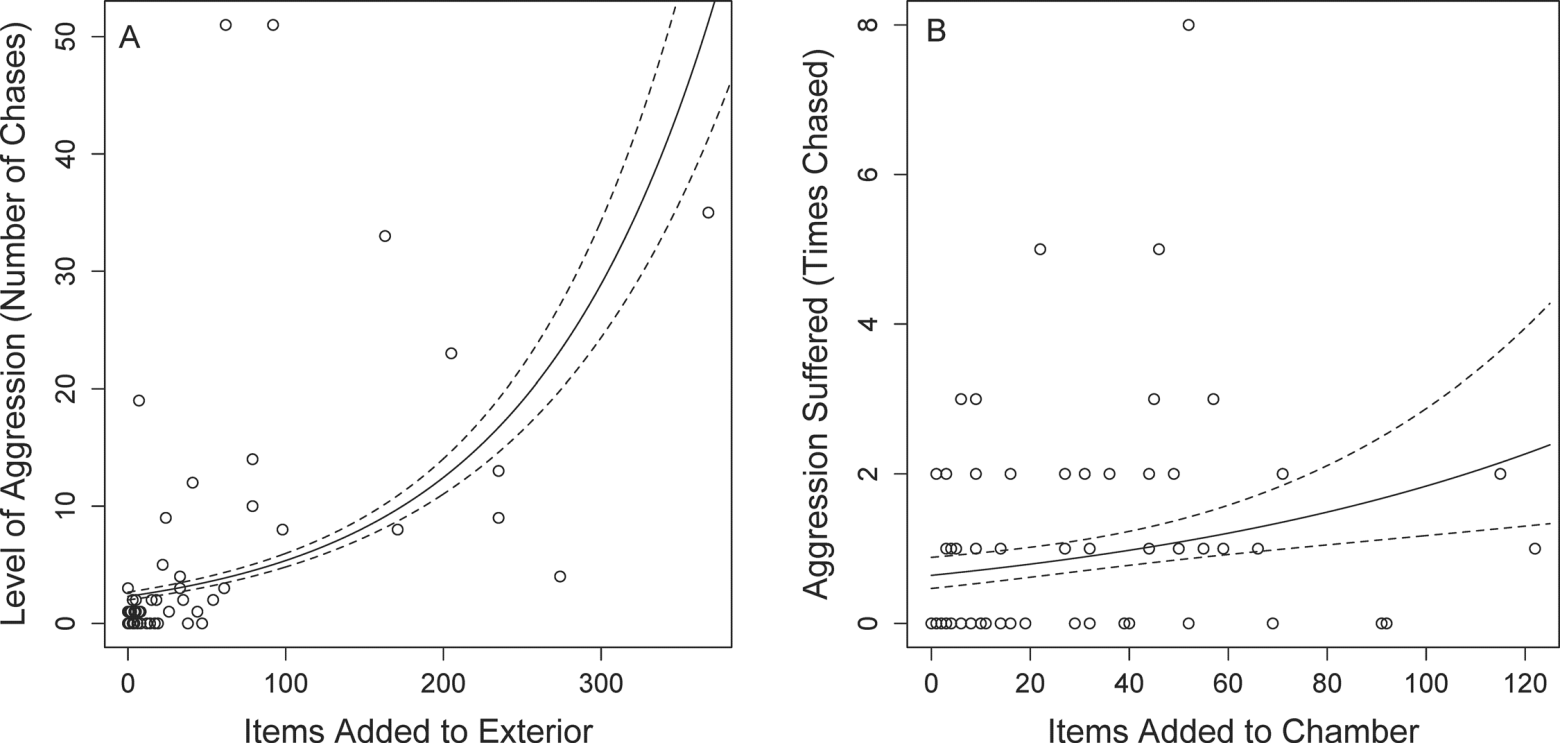
(A) Positive association between the number of items added to the thatch of the nest and the number of chases an individual performed. (B) Positive relationship between the number of items added to a nest chamber and the aggression suffered by the individual. In both plots the solid line is the predicted line from the generalized linear mixed model while the dashed lines represent the 95% confidence intervals around the line.

**Fig 2:**
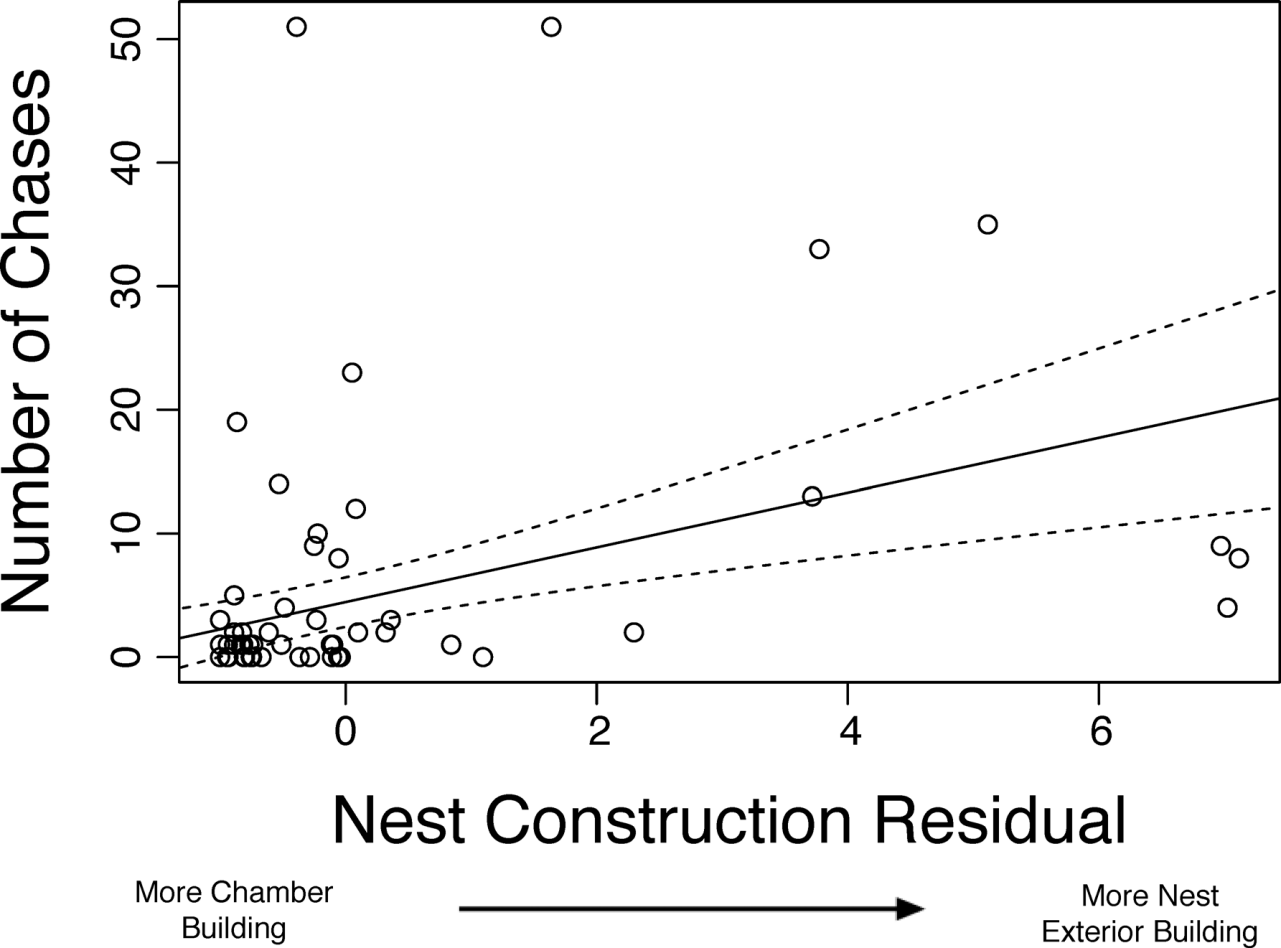
Positive association between the nest construction residual and the number of chases an individual performed. The nest construction residuals are generated from a linear model that predicts number of items inserted into nest thatch based on the number of items inserted into nest chambers. Positive residuals represent weavers that inserted more items into the nest thatch relative to the expectation from the regression. The solid line is the predicted line from the linear model while the dashed lines represent the 95% confidence interval around the line.

We recorded the behavior of 83 sociable weavers and found that aggression, i.e. the number of times an individual chased other individuals, was significantly and positively associated with the number of items inserted into the nest thatch according to the GLMM (β = 0.07 ± 0.01, t_3240_ = 4.89, p < 0.001, Fig 1A, Table 1, N = 342 identified aggressive chases, N = 2740 instances of thatch nest construction). The number of chases an individual performed was positively associated with number of items inserted into the chambers in the GLMM (β = 0.09 ± 0.03, t_3422_ = 2.76, p = 0.005). Additionally, wing length was the only morphological variable associated with aggression, though birds with larger beak depths trended towards being more aggressive (Table 1). Surprisingly, sex was not a predictor of aggression (β = -0.009 ± 0.02, t_3420_ = -0.42, p = 0.68). The full model (including random factors) explained ~51% of the variation, with the independent variables explaining 17% of the variation (see Table 1 for list). Since both external nest construction and chamber maintenance predict aggression, we investigated the relationship between these two variables and how they influenced aggression. There was a significant positive relationship between thatch nest construction and interior nest construction in a linear model (β = 0.20 ± 0.03, t_3747_ = 6.6, p < 0.001), though the relationship did not explain much variation (R^2^ = 0.04). We therefore used the residuals from this model to predict aggressive behavior. In a second linear model we found that individuals with more positive residuals, i.e. individuals that devote significantly more nest construction effort to inserting items into the communal thatch relative to nest chambers, were more aggressive (β = 0.01 ± 0.001, t_3387_ = 10.8, p < 0.001, Fig 2). We also found that aggressive behavior is repeatable within individuals, r = 0.406 (95% C.I. = 0.245–0.506) according to repeatability formula specified by Nakagawa and Schielzeth [51].

**Table 1:** The generalized linear mixed model predicting the number of times an individual chased other nest mates. P-values represent whether regression coefficient is significantly different from zero.

Variable	Coefficient	Std. Error	t-value	P-value
Condition	0.13	0.15	0.86	0.39
Wing Length	0.43	0.17	2.52	0.01
Tarsus	0.12	0.25	0.48	0.63
Beak Depth	0.95	0.52	1.83	0.07
Beak Width	-0.69	0.44	-1.56	0.12
Items Added to Thatch	0.07	0.01	4.88	<0.001
Items Inserted Into Nest Chamber	0.09	0.03	2.76	0.005
Age Class	-0.01	0.03	-0.386	0.71

In the second GLMM the number of times an individual was chased was weakly, but positively associated with the number of items that they insert into their nest chamber (β = 0.23 ± 0.07, t_2149_ = 3.36, p < 0.001, Fig 1B, Table 2, N = 73 identified instances of suffering aggressive chase, N = 2005 instances of chamber maintenance). The number of times an individual was chased was also negatively correlated with beak width (β = -0.96 ± 0.34, t_74.7_ = 2.83, p = 0.005), while no other variables explained the variation in the aggression suffered by an individual, though individuals with shorter wings trended towards suffering more aggression (Table 2). Again, sex was not a predictor of suffering aggression (β = -0.003 ± 0.005, t_3421_ = -0.56, p = 0.57). Suffering aggression was not repeatable within individuals (r = 0.01, Table 3). The full model predicting the amount of aggression suffered explained ~25% of the variation, with the independent variables explaining 7% of the variation (see Table 2 for list of independent variables).

**Table 2:** The linear mixed model predicting the number of times an individual was chased by other nest mates. P-values represent whether regression coefficient is significantly different from zero. Note that the variable indicating sex represents an analysis with NAs removed. In that analysis sex was not a significant predictor of suffering aggression, so we removed that factor and analyzed the full dataset with the remaining factors.

Variable	Coefficient	Std. Error	t-value	P-value
Condition	0.08	0.11	0.71	0.48
Wing Length	-0.21	0.11	-1.85	0.06
Tarsus	-0.05	0.19	-0.29	0.77
Beak Depth	0.36	0.40	0.92	0.36
Beak Width	-0.96	0.33	-2.83	0.005
Items Added to Thatch	0.06	0.04	1.93	0.20
Items Inserted Into Nest Chamber	0.23	0.07	3.36	<0.001
Age Class	-0.006	0.004	-1.04	0.3

**Table 3:** Repeatabilities and the 95% confidence interval around the estimate. Repeatabilities were calculated using the “rptR” package in R using an linear model with MCMC fitting.

Behavior	Repeatability	95% Confidence Interval
Items Added to Chamber	0.308	0.252–0.377
Items Added to Thatch	0.109	0.074–0.141
Aggressive Chases	0.406	0.245–0.506
Number of Times Chased	0.01	0–0.046

**Fig 3:**
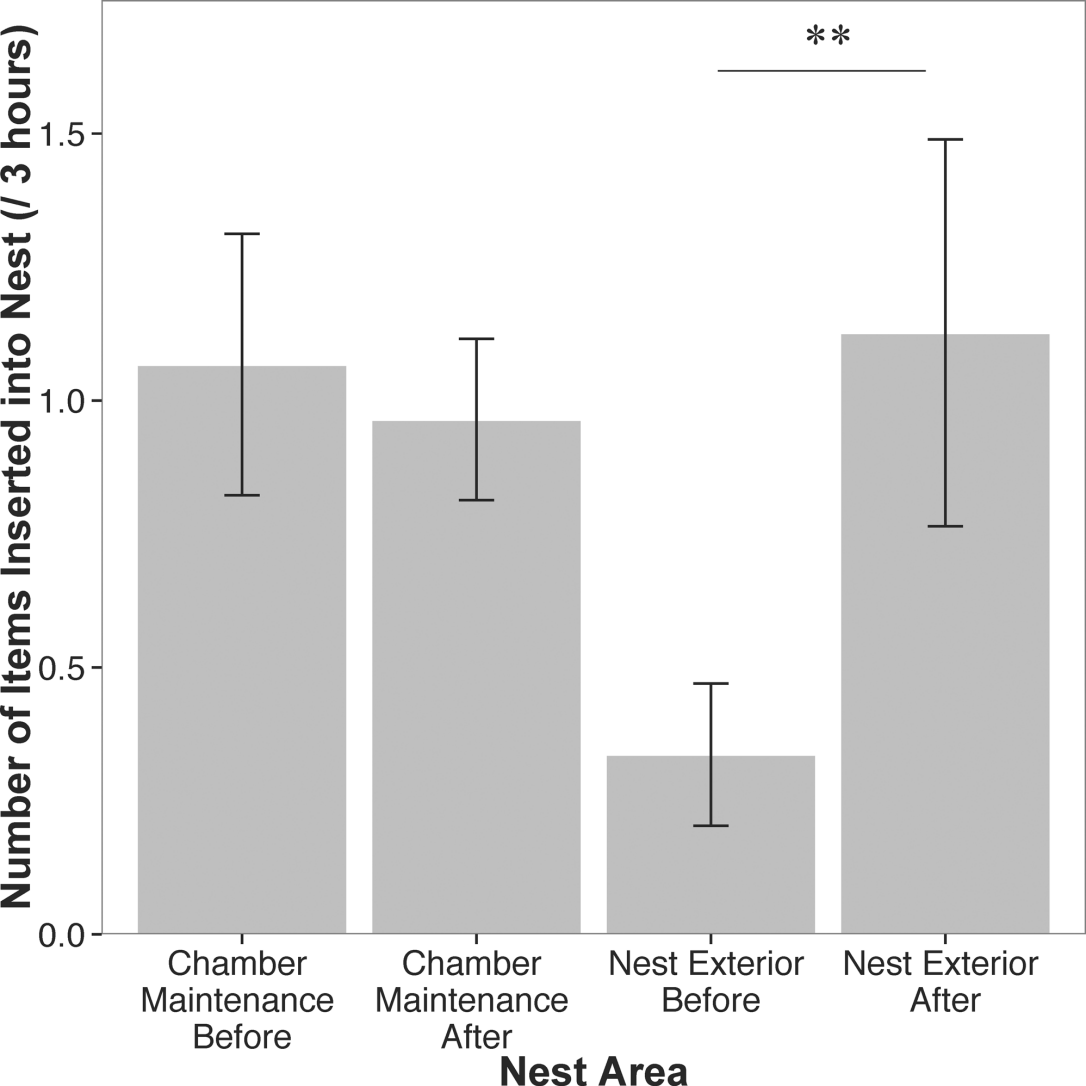
Mean number of items inserted into the nest. Left two bars represent items inserted into the nest chamber before and after aggression, respectively. The right two bars represent the number of items inserted into the nest thatch before and after aggression, respectively. There is a significant increase in items inserted into the nest thatch after an individual suffers aggression. Error bars represent ± 1 S.E.M.

**Fig 4:**
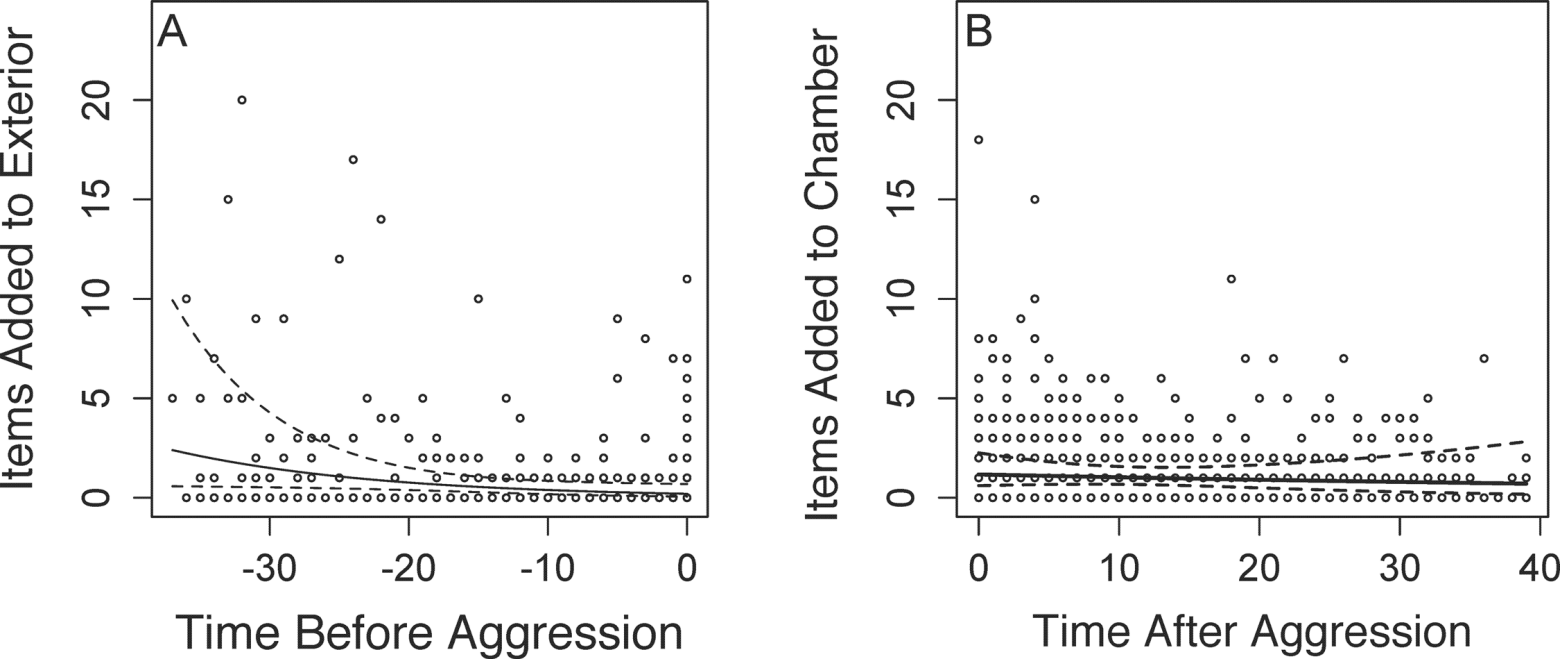
(A) Negative relationship between the number of items added to the thatch of the nest and the number of observation blocks before suffering aggression. (B) Negative association between the number of items added to a nest chamber and the time after suffering aggression. Each unit represents one observation block (3 hours), and we completed two observation blocks per day. Therefore 2 units on these axes represent 24 hours. In both plots the solid line is the predicted line from the linear model while the dashed lines represent the 95% confidence interval around the line.

When combining across all the time points before or after aggression, we found that sociable weavers that suffer aggression contribute significantly more to cooperative nest construction after aggression compared to before being chased according to the linear mixed model comparing thatch construction before and after a chase (F = 8.13, p = 0.008, Fig 3). We note that this analysis was performed on the subset of individuals that were chased at least once (33 out of 83 individuals). We therefore investigated how behaviors changed leading to an aggressive bout using a final GLMM that examined how nest construction changed before and after a chase. There was a negative relationship between the time before suffering aggression and the number of items added to the nest thatch, i.e., as the time before suffering aggression approaches, individuals significantly decreased the number of items they added to the nest thatch (β = -0.04, Std. Error = 0.009, z = -43.9, p < 0.001, Fig 4A). After suffering a bout of aggression individuals show a slight but significant reduction in their chamber maintenance as time progresses after aggression (β = -0.02, Std. Error = 0.003, z = -5.5, p < 0.001, Fig 4B).

## Discussion

Sociable weavers that spend more time cooperatively constructing the thatch of the nest are also more aggressive in terms of chasing other individuals away from the nest (Fig 2). In other words, individuals that preferentially build the communal thatch, rather than maintain individual nest chambers, were significantly more aggressive. These results are consistent with the hypothesis that cooperative individuals that are at the most risk of exploitation should be selected to minimize the exploitation of their cooperative output [38]. There was a weak but positive association between maintaining a nest chamber (Fig 1B) and suffering aggression, and individuals were more likely to be chased when they had decreased nest construction of the communal thatch prior to being chased (Fig 4). This combination of results suggests that reductions in thatch construction may be targeted for aggression in sociable weavers though we emphasize that these results need to be experimentally verified. The chased individuals are not evicted, however, as we observe these same individuals at the nest in following observation periods (G.M. Leighton, pers. obs.). Individuals respond to aggression by significantly increasing construction of the communal thatch (Fig 3), and these results are consistent with the expectations of punishment [38]. After suffering aggression individuals significantly increase cooperative maintenance of the communal thatch. However, the only behavioral predictor of suffering aggression was performing chamber maintenance. This does not fit the expectations of punishment because we would have predicted that those individuals that performed no nest construction of any type would receive the most aggression. We therefore suspect that this system does not meet all expectations of the original model of punishment *sensu stricto*.

In contrast, a more plausible alternative to the punishment model [26] is the pay-to-stay model [52, 53]. The pay-to-stay model suggests that subordinates cooperate to avoid aggression or eviction from the group. Several results from this study comport well with the predictions of the pay-to-stay model. First, the cooperative and aggressive individuals may be the dominant individuals given the positive association between wing length and aggression (Table 1). Indeed such an association between wing length and dominance has been found in other avian taxa [54], though this relationship needs to be verified experimentally in the field. The aggressive individuals may be directing aggression towards smaller subordinates, as suggested by the increased aggression towards individuals with smaller beak widths (Table 2). If dominants are aggressive to induce thatch construction, and if the subordinates reside close to the dominants within the nest, then this increased nest construction would benefit the dominants as individuals typically build in areas of the nest where they reside [33, 38]. The presence of cooperative subordinates could be beneficial enough to dominants to allow subordinates to stay in the group despite the increased cost of resource sharing. The increased cooperation in response to aggression (Fig 2) is also a prediction of the pay-to-stay hypothesis as individuals increase cooperative behavior due to the threat of permanent eviction [55]. The reduction in nest construction preceding an attack (Fig 4A) suggests that subordinates that maintain a high level of thatch construction may be due to “pre-emptive appeasement” [11]. If individuals devote too much construction towards the chamber (Fig 1B) or reduce construction overall (Fig 4A), dominant individuals use aggression to redirect individuals to thatch construction. The pay-to-stay model is therefore a more likely explanation for this behavior than the punishment model given the series of results that support the pay-to-stay model.

Importantly, some of the results we present may function simultaneously to establish dominance. For instance, aggressive individuals had significantly longer wings (Table 1, though age did not explain wing length, t_83_ = 1.4, p > 0.1), while individuals that had significantly narrower beaks suffered significantly more aggression (Table 2). Other morphological variables did not predict aggression or suffering aggression (Tables 1 and 2), and the morphological variables were not strongly correlated with each other (all r < 0.3). These results suggest that it was not uniformly larger birds being aggressive towards smaller birds. One possibility is that aggressive chases evolved in sociable weavers in the context of establishing dominance; but if aggression now also induces nest construction, it may also be maintained because of its effects in this cooperative context.

One interesting aspect of these results is that aggression is associated with thatch construction and nest chamber maintenance (Table 1). This may suggest that this suite of behaviors may be explained by overall activity; however, we found that the birds that invested relatively more in thatch construction relative to chamber maintenance were especially aggressive (Fig 2). This relationship is similar to that in the cooperatively breeding cichlid, *N*. *pulcher*, where dominants invest more in territory maintenance but use aggression to recruit subordinates to increase cooperation [55], another system where the pay-to-stay mechanism maintains cooperation.

A limitation of our study is that we observed three nests in a single tree. However, the behavior of individuals between nests is consistent over time and when comparing our results to the same behavior of sociable weavers at other study sites [33]. For example, individual repeatability in sociable weavers is relatively high [37, 56]; and the external nest construction observed in sociable weavers is similar across studies [32, 33, 37]. Importantly, focused behavioral observation of sociable weavers is often necessary to delimit relationships between behavior and other variables (e.g. relatedness and cooperative output, [33]). As our results are consistent with some expectations of the punishment model future work should both confirm the basic trends observed in this study while also investigating experimentally testing hypotheses regarding aggression and punishment.

One unexplained aspect of aggression inducing cooperation in this system is that many sociable weavers do not perform any thatch nest construction [32, 37]; in this study we found that 29 of the 83 individuals (~35%) never constructed the thatch of the nest. Indeed, other studies of sociable weaver nest construction suggest that a minority of birds never invest in thatch construction [37]. A subset of sociable weavers is not observed at the nest during the day [33], and therefore these individuals can not be chased, however we suspect that this group that returns to the nest in the evening to roost. Although one possibility is that these individuals have alternative options and can thus avoid aggression, we suspect these individuals are spending the entire day foraging due to potential caloric deficits that may result from roosting near the edge of a nest [34, 35].

Recent work in vertebrate societies suggests that individuals attend to the behavior of conspecifics within the group, and behavioral manipulations have demonstrated that aggression or punishment are critical components of many animal societies [11–13, 20, 25]. However, in these systems there is less active punishment as compared to sociable weavers, where individuals seem to actively and constantly use aggression. As predicted by the pay-to-stay model, the result of this aggression often results in the increase of cooperative behavior of group mates. This is similar to previous work in naked mole-rats (*Heterocephalus glaber*) where queens shoved inactive workers to induce behaviors that benefit the colony [57]. In contrast to naked mole-rats, however, there is no single individual in a sociable weaver nest that performs all of the aggressive behavior. This aggressive behavior in sociable weavers may therefore be most similar to the behavior of dominants in cooperatively breeding cichlids that induce cooperative investment in communal resources from subordinates [55].

In addition to the concern about the cost of enacting aggression [58], a second concern of models where aggression is used to induce cooperation, e.g. punishment or pay-to-stay, is that directed aggression requires individuals to have a neurological system capable of attending to the behavioral output of conspecifics [59]; in the case of sociable weavers this would suggest that individuals would have to observe potentially dozens of other weavers and their nest construction. Indeed, monitoring the entire behavioral output of others when that behavior relies on items like twigs or grasses leads to relatively inaccurate assessments of the behavior in question [60]. We therefore do not find it likely that sociable weavers actively quantify the nest construction of conspecifics. Instead, we hypothesize that aggression could also be effective if individuals rely on simple rules to mete out aggression. For instance, individuals may simply chase an individual if they observe the individual insert items into a nest chamber more than once in a given period of time. Since simple rules are used for performing behaviors in other social bird species [61], we argue that simple rules could direct aggressive behavior and result in the trends we present here. These presence of simple rules does not necessarily mean that a species has limited cognition and would require further studies to determine if simple rules are correlated with limited cognition.

We present evidence that suggests that aggression may be utilized to maintain cooperative nest construction in sociable weavers, though our results do not comport entirely with previous models of punishment. Instead, our results more closely align with expectations from the pay-to-stay model, where dominant individuals induce cooperative behavior in subordinates. More work is necessary to determine whether there is a causal relationship between reduced thatch construction and aggression. This would require experimental manipulations of cooperative nest construction, for instance, a future test that reduces thatch construction via removal of individuals, and assays for changes in aggressive behavior would determine conclusively if subordinate sociable weavers are paying to stay with cooperative nest construction.
