# Correction: Sociable Weavers Increase Cooperative Nest Construction after Suffering Aggression

**DOI:** 10.1371/journal.pone.0154470

**Published:** 2016-04-21

**Authors:** The PLOS ONE Staff

The second author’s last name is incorrectly displayed in the citation. The publisher apologizes for the error.

The correct citation is: Leighton GM, Vander Meiden LN (2016) Sociable Weavers Increase Cooperative Nest Construction after Suffering Aggression. PLoS ONE 11(3): e0150953. doi:10.1371/journal.pone.0150953

## References

[1] Leighton GM, Meiden LV (2016) Sociable Weavers Increase Cooperative Nest Construction after Suffering Aggression. PLoS ONE 11(3): e0150953 doi:10.1371/journal.pone.0150953 26982704 10.1371/journal.pone.0150953 PMC4794138

